# Stochastic Simulation of Delay-Induced Circadian Rhythms in *Drosophila*

**DOI:** 10.1155/2009/386853

**Published:** 2009-06-15

**Authors:** Zhouyi Xu, Xiaodong Cai

**Affiliations:** 1 Department of Electrical and Computer Engineering, University of Miami, Coral Gables, FL 33124, USA

## Abstract

Circadian rhythms are ubiquitous in all eukaryotes and some prokaryotes. Several computational models with or without time delays have been developed for circadian rhythms. Exact stochastic simulations have been carried out for several models without time delays, but no exact stochastic simulation has been done for models with delays. In this paper, we proposed a detailed and a reduced stochastic model with delays for circadian rhythms in *Drosophila* based on two deterministic models of Smolen et al. and employed exact stochastic simulation to simulate circadian oscillations. Our simulations showed that both models can produce sustained oscillations and that the oscillation is robust to noise in the sense that there is very little variability in oscillation period although there are significant random fluctuations in oscillation peeks. Moreover, although average time delays are essential to simulation of oscillation, random changes in time delays within certain range around fixed average time delay cause little variability in the oscillation period. Our simulation results also showed that both models are robust to parameter variations and that oscillation can be entrained by light/dark circles. Our simulations further demonstrated that within a reasonable range around the experimental result, the rates that *dclock* and *per* promoters switch back and forth between activated and repressed sites have little impact on oscillation period.

## 1. Introduction

Almost all living organisms, including animals, plants, fungi, and cyanobacteria, exhibit daily periodic oscillations in their biochemical or physiological behavior, which are known as circadian rhythms [1–7]. The mechanism of circadian oscillation has been an extensive research topic in the last three decades. It has been found that circadian rhythms in fact are determined by oscillatory expression of certain genes [8,9]. Specifically, circadian clocks consist of a network of interlocked transcriptional-translational feedback loops formed by a number of genes [2]. In *Drosophila*, transcription of *per* and *tim* genes is activated by a heterodimer consisting of two transcriptional activators dCLOCK and CYCLE [10–13]. The PER protein in turn binds to the dCLOCK-CYCLE heterodimer, which inhibits the DNA binding activity of the dimer, thereby repressing the transcription of *per* and *tim* [11–14]. While this forms a negative feedback loop, there is also a positive feedback loop, in which PER and TIM activate dCLOCK synthesis by binding dCLOCK and relieving dCLOCK's repression of dclock transcription [15,16].

Several mathematical models have been proposed for circadian oscillation in *Drosophila* [12,14,17–22]. The models of Smolen et al. [12,14] introduce time delays in the expression of *dclock* and *per* genes, while other models do not have such delays. Numerical simulations using ordinary differential equations (ODE) show that all these models can produce circadian oscillations. In particular, times delays were found to be essential for simulation of circadian oscillations with the model of Smolen et al. [12,14].

Since there is significant stochasticity in gene expression arising from fluctuations in transcription and translation [23–25], it is desirable to simulate circadian oscillations in the presence of noise. Toward this end, several stochastic models were proposed [4,26–29], and Gillespie's stochastic simulation algorithm (SSA) [30,31] was employed to simulate circadian oscillations. All these stochastic models [4,26–29] do not include time delays. In order to reflect the noise in gene expression, Smolen et al. used two approximate stochastic simulation methods to simulate circadian oscillation based on their models with delays [12,14]. However, their models lumped transcription and translation into one single process and did not model the process that dCLOCK binds to or dissociates with *dclock* and *per* genes to activate or inhibit transcription. Since transcription is a major source of intrinsic noise [23,24], the approximate stochastic simulation of Smolen et al. may underestimate the effect of noise. Li and Lang [32] used similar approximate stochastic simulation methods to simulate reduced model of Smolen et al. [14], but with an emphasis on the noise-sustained oscillation in the region of parameter values where the deterministic model predicted no oscillation. Currently, no exact stochastic simulation has been done for circadian rhythm models with random delays, partially due to the fact that Gillespie's SSA cannot handle delays in certain reactions.

Recently, we developed an exact SSA algorithm for systems of chemical reactions with delays [33]. The goal of this paper is to apply this exact SSA to simulate circadian oscillations in *Drosophila* using a model with time delays and to investigate the effects of noise and random time delays on circadian oscillations. We first develop two stochastic models with random delays for circadian oscillations in *Drosophila* based on the two deterministic models of Smolen et al. [12,14]. Using our exact SSA, we then simulate free-running circadian oscillation under constant darkness. Our simulations demonstrate that both models can produce sustained oscillations. The variability in oscillation period is very small although the variability in oscillation peaks is considerably large. In particular, although time delays are essential to oscillation, random fluctuations in time delays do not cause significant changes in oscillation period as long as the average delays are fixed. Our simulations also showed that circadian oscillations of both models are robust to parameter variations. The entrainment by light was also simulated for both models, yielding results consistent with experimental observations. To see the effect of transcription noise, we also run simulations with different values for the rate that dCLOCK binds or unbinds to *per* and *dclock* genes.

## 2. Methods

### 2.1. The Detailed Model of Circadian Oscillation with Time Delays

#### 2.1.1. Model Description

**Figure 1:**
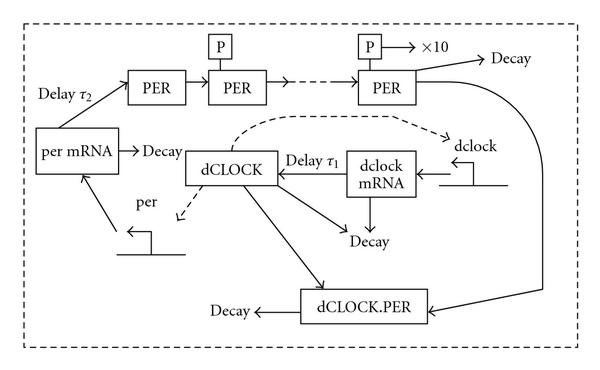
**Schematic of the detailed model for circadian oscillators in *Drosophila***.

We develop a stochastic model for the *Drosophila* circadian oscillator based on the deterministic model of Smolen et al. [12], which is depicted in Figure 1. In Smolen's model, transcription of *dclock* gene is repressed by dCLOCK protein after a time delay of  [11,13]. dCLOCK activates the synthesis of PER protein with a time delay of . PER is then phosphorylated [34], and unphosphorylated and phosphorylated PER can bind to dCLOCK thereby relieving dCLOCK's repression of *dclock* transcription. It was reported that PER undergoes multiple and sequential phosphorylation [34], but exact times of phosphorylation are unknown. Following Smolen et al. [12], we assumed that PER can be phosphorylated up to 10 times. Although the TIM gene also plays an important role in circadian rhythm, Smolen et al. [12] used a single "lumped" variable, PER, to represent both PER and TIM, since the time courses of PER and TIM proteins are similar in shape and largely overlap. Smolen et al. [12] characterized the circadian oscillator in *Drosophila* using 23 ordinary differential equations (ODEs). We first convert these 23 ODEs into 46 chemical reactions. Smolen et al. [12] lumped transcription and translation of *dclock* and *per* into one single step. They did not model the process that dCLOCK binds to and dissociates with *dclock* gene and *per* gene. Since this binding and unbinding processes, transcription, and translation are major sources of intrinsic noise [35–38], we model these processes explicitly. Our stochastic model, containing 29 molecular species in Table 1, is featured with 54 reactions in Table 2 which include 44 reactions converted from Smolen's ODE and 10 new reactions.

**Table 1:** Detailed stochastic model for the  circadian oscillator.

Rid	Reaction	Rate ()
1	dclockg dclockg + dclockm	
2	dclockm	
3	dclockm dclockm + dCLOCK (delay )	
4	dCLOCK	
5	dclockg + dCLOCK dclockg.dCLOCK	
6	dclockg.dCLOCK dclockg + dCLOCK	
7	dCLOCK + perg perg.dCLOCK	
8	perg.dCLOCK dCLOCK + perg	
9	perg.dCLOCK perg.dCLOCK + perm	
10	perm	
11	perm perm + (delay )	
	,	
22		
	dCLOCK + dCLOCK.	
	dCLOCK. dCLOCK.,	
	dCLOCK.,	
54	dCLOCK.	

*See text for detailed descriptions.

**Table 2:** Molecular species.

Species	Description
dclockg	gene
dclockm	mRNA
dCLOCK	dCLOCK protein
dclock.dCLOCK	gene bounded by dCLOCK protein
perg	gene
per.dCLOCK	gene bounded by dCLOCK protein
perm	mRNA
,	PER protein with phosphorylations
dCLOCK.,	Complex of dCLOCK and PER with phosphorylations

Reaction  represent transcription of  gene, degradation of  mRNA, translation of  mRNA, and degradation of dCLOCK protein, respectively. Reaction 5 models the process that dCLOCK protein binds to  gene and reaction 6 represents dissociation of dCLOCK with . Reaction 7 and 8 specify the event that dCLOCK binds to and dissociates with  gene. Reactions 9, 10, and 11 represent transcription of  gene after it is activated by dCLOCK, degradation of  mRNA, and translation of  mRNA, respectively. Reactions  represent the phosphorylation of PER and reaction 22 represents the degradation of PER. Reactions  represent the association of dCLOCK with PER at different levels of phosphorylation. Reactions  describe the phosphorylation of dCLOCK and  () heterodimer. Reactions  represent the degradation of dCLOCK and  () heterodimer.

#### 2.1.2. Parameter Estimation

Each reaction is associated with a reaction probability rate constant, , which determines the probability that a specific reaction occurs in an infinitesimal time interval. The probability rate constant  of a specific reaction can be calculated from conventional rate constant  as follows:  for a monomolecular reaction,  for a bimolecular reaction with two different reactants, and  for a bimolecular reaction with one reactant [39], where , and  is the Avogadro constant and  is the system volume. We assume that a lateral neuron in  is a sphere of a radius around 6  [14,40], which results in a volume  L. As many other existing models [12,14,41], we do not separate nuclear and cytoplasmic compartments. We retain most parameter values from Smolen's et al. [12] including  and . The remaining 10 parameters, , , , and , are determined in our simulation. In the following, we describe 54 reactions and how each probability rate constant was determined.

We assume that there are two copies of  genes and thus the initial value for the number of molecules of  in Table 1 is 2. Since no experimental reports are available for transcription rate , we choose  , which is close to the value used in a previous computational model [41]. The degradation rate of  mRNA  is calculated as , where  is the half-life of  mRNA. Hardin et al. [42] shows that mRNA of periodic genes in  has short half-life, varying from order of minutes to tens of minutes. Lin et al. [43] shows that  mRNAs vary considerably in half-life from tens of minutes to more than 10 hours. Here we assume that the average half-life of  mRNA is 65 minutes, which is 1.08 hours and thus  is  . The synthesis rate of dCLOCK protein was chosen to be  nM  in the model of Smolen et al. [12], which equivalently is  molecules per hour. In our model, the average dCLOCK synthesis rate is  molecules per hour since we assume the number of molecules of  in Table 1 is 2. Letting , we get  . Since transcription rate has a significant impact on the noise [23,24], we tested the sensitivity of simulation results to . Increasing or decreasing  two times while fixing the ratio of  only causes negligible change in the mean and standard error (SE) of period and peaks (data not shown). The rate  is calculated as  [12], where  nM ,  nM, and  is the concentration of total dCLOCK given by (1)

Here  represents the concentration of the species in the bracket. A time delay  is included in reaction 3 accounting for time needed for transcription, translation, and other potential mechanisms for activating the transcription of . Smolen et al. chose  to be a deterministic number equal to 5 hours [12]. Taking into account uncertainty in this delay, we choose  as a random variable uniformly distributed in the interval (4h–6h).

In reaction 5, dCLOCK binds to the E-box of  [11,13], but there is no experimental report on the values of  and the dissociation rate . However, the dissociation rate of myogenin protein with the E-box of E12 gene was reported to be   [44]. Therefore, we choose   . The equilibrium constant  of reactions 5 and 6 is equal to the Michaelis constant  in [12] that describes the regulation of dCLOCK synthesis by dCLOCK and was chosen to be 1 nM [12]. Using this equilibrium constant, we calculate  to be  . Reactions 7 and 8 specify the event that dCLOCK binds to and dissociates with  gene. The equilibrium constant  of reactions 7 and 8 is equal to the Michaelis constant  in [12], which was 1 nM. This Michaelis constant reflects the regulation of PER synthesis by dCLOCK. After choosing ,  is calculated from the equilibrium constant  as  .

The transcription rate of  gene  is chosen to be 20 , and the degradation rate of  mRNA  is calculated as   from the half-life of  mRNA which was estimated to be 2 hours [42,43]. Also, we assume that there are two copies of  gene, and thus, the initial value for the number of molecules of *perg* in Table 1 is 2. The synthesis rate of PER protein was chosen to be  nM  in the model of Smolen et al. [12], which equivalently is  molecules per hour. In our model, the average PER synthesis rate is  molecules per hour. Letting , we got  . Similar to , we also tested the sensitivity of simulation results to . Increasing or decreasing  two times while fixing the ratio of  only causes negligible change in the mean and standard error (SE) of period and peaks (data not shown).

A delay  is introduced in reaction 11. This time delay accounts for the time needed for the transcription and translation of  gene. Smolen et al. [12] chose  to be 8 hours. However, the total time needed for transcription and translation maybe less than 8 hours [45] and also there may be some fluctuations in . Therefore, we chosen  as a random variable with mean 6 hours, uniformly distributed in (4.8h–7.2h).

Reactions  represent the phosphorylation of PER, whose probability rate constants are all equal to  [12], where  nM ,  nM and  is the concentration of all forms of PER with less than 10 phosphorylations given by (2)

Reaction 22 represents the degradation of PER and  is equal to , where  nM  and  nM [12].

Reactions  represent the association of dCLOCK with PER at different levels of phosphorylation. The deterministic rates for all these reactions are 30 n and thus the probability rate constants are  , . Reactions  describe the phosphorylation of dCLOCK and  () heterodimer and we have , . Reactions  represent the degradation of dCLOCK and  () heterodimer. We have ,  and , where  and  are given earlier.

### 2.2. The Reduced Model of Circadian Oscillation with Time Delays

#### 2.2.1. Model Description

Smolen et al. [14] also simplified their detailed model described earlier by removing the phosphorylation of PER. This reduced model was characterized by 2 ODEs. We first convert these 2 ODEs into 4 chemical reactions. Again, we explicitly model the binding and unbinding of dCLOCK to  and  genes, as well as the transcription and translation of  and  genes. Our reduced stochastic model consists of 9 molecular species and 14 reactions specified in Table 3. Comparing with reactions in Tables 1 and 3, we see that reduced model is obtained by removing reactions related to phosphorylation of PER and phosphorylated PER. Similarly to the detailed model, we retained most parameter values from Smolen et al. [14], including  and . The parameters not presented in Smolen's reduced model are determined and explained in the following subsection.

**Table 3:** Reduced stochastic model for the  circadian oscillator.

Rid	Reaction	Rate ()
1	dclockg dclockg + dclockm	
2	dclockm	
3	dclockm dclockm + dCLOCK (delay )	
4	dCLOCK	
5	dclockg + dCLOCK dclockg.dCLOCK	
6	dclockg.dCLOCK dclockg + dCLOCK	
7	dCLOCK + perg perg.dCLOCK	
8	perg.dCLOCK dCLOCK + perg	
9	perg.dCLOCK perg.dCLOCK + perm	
10	perm	
11	perm perm + PER (delay )	
12	PER	
13	dCLOCK + PER dCLOCK.PER	
14	dCLOCK.PER	

#### 2.2.2. Parameter Estimation

The rate  is chosen to be 10  which is slightly lower than that in the detailed model. This is because the synthesis rate of dCLOCK protein in the reduced model of Smolen et al. [14] was  nM , which is smaller than  in the detailed model. The rate   is the same as that in the detailed model. Letting  equal to , we calculate  . The rate  is the same as that in the reduced model of Smolen et al. [14], equal to 0.5 .

The unbinding rate of dCLOCK to  gene, , is chosen identical to that in the detailed model. The equilibrium constant , which is equal to the Michaelis constant  in [14] that describes the regulation of dCLOCK synthesis by dCLOCK, was chosen to be 0.1 nM [14]. Using this equilibrium constant, we calculate  to be 1.44 . Similarly,  is the same as that in the detailed model. The equilibrium constant , which is equal to the Michaelis constant  in [14] that describes the regulation of PER synthesis by the transcriptional activators dCLOCK, is chosen to be 0.3 nM [14]. Then we calculate  to be 0.48 .

The transcription rate of  gene  is chosen to be 10 , which is lower than that in the detailed model, because the synthesis rate of PER protein in the reduced model of Smolen et al. [14] was  nM  which is smaller than that in the detailed model. The degradation rate of  mRNA is again  . Letting  equal to , we calculate  as  . The degradation rate of PER  is the same as that in the reduced model of Smolen et al. [14], equal to 0.5 . The degradation rate of dCLOCK and PER complex is  , identical to that in the detailed model and we have .

Time delays  and  are chosen as follows. As the effective delay contributed by PER phosphorylation is incorporated into  and , , and  should be longer than those in the detailed model. Therefore we chose  and  uniformly distributed in the time interval (5h–9h) and (7h–11h), respectively.

### 2.3. Stochastic Simulation

Gillespie's SSA [31] is often employed to simulate the stochastic dynamics of genetic networks [23,46]. However, Gillespie's SSA cannot deal with delays in certain reactions. Recently, we developed an exact SSA for systems of chemical reactions with delays [33], which can handle both deterministic and random delays. We use this exact SSA to simulate the dynamics of the systems described in Tables 1 and 3.

### 2.4. Data Analysis

Customized MATLAB Software (Mathworks Inc.) was written to analyze data generated from stochastic simulations, for example, to calculate the mean and SE of protein levels, to identify the peaks of dCLOCK and PER during oscillation, and to calculate the peak amplitudes. Oscillation periods were calculated using the short-time Fourier transform (STFT) method [47]. Specifically, Fourier transform was applied to protein levels of dCLOCK and PER within a time window of 70 hours, after the mean level was subtracted. The largest peak at a non-zero frequency was identified as the oscillation frequency within the time window and the period of the oscillation is the inverse of the oscillation frequency. Note that the maximum period that can be identified by the STFT is 35 hours since a time window of 70 hours was used.

## 3. Results

### 3.1. Simulation of Oscillation in the Presence of Noise

**Figure 2:**
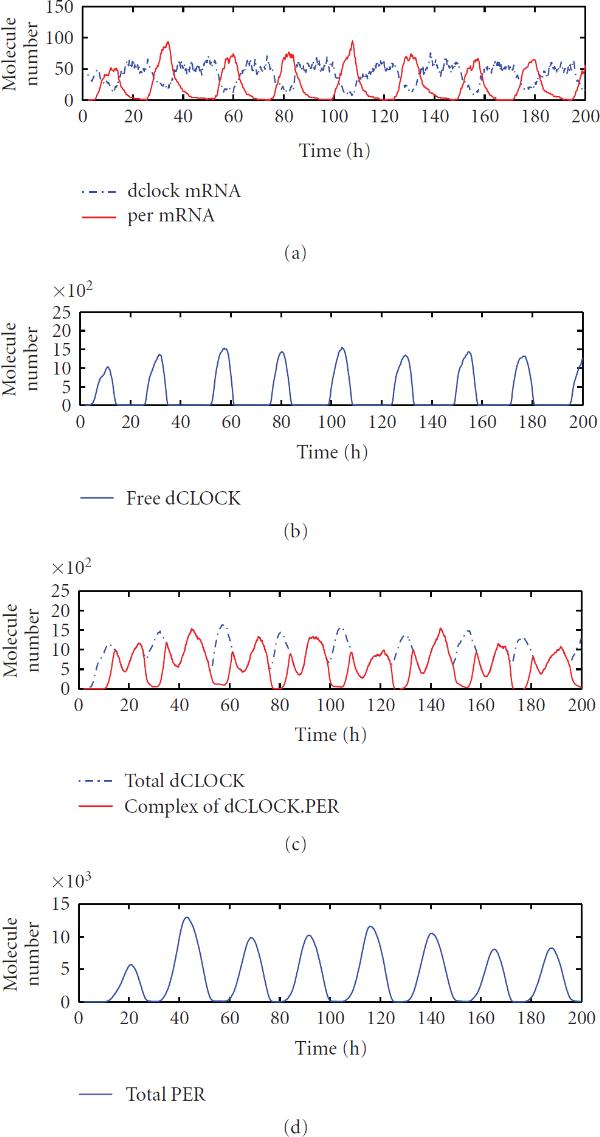
One trajectory of  and  mRNA, free dCLOCK, total dCLOCK, dCLOCK.PER complex and total PER for the detailed stochastic model in constant darkness.

We first ran simulations using the detailed model. Figure 2 depicts one trajectory of the number of molecules of  and  mRNA, free dCLOCK protein, the total number of molecules of dCLOCK that includes dCLOCK, dclock.dCLOCK, per.dCLOCK, and dCLOCK., , in Table 2, the total number of molecules of dCLOCK.PER which includes dCLOCK., , in Table 2, and the total number of PER protein that includes  and dCLOCK., , in Table 2. We here simulated free-running rhythms in constant darkness. Figure 2 clearly shows oscillations of the levels of mRNA and protein despite some random fluctuations. It is seen that  and  oscillations are almost in antiphase with each other, which is consistent with the experiment observations [45,48,49]. It appears that there are more fluctuations in mRNA levels than the corresponding protein levels. This is due to the fact that the number of mRNA molecules is much lower than those of proteins. Even though the shape of free dCLOCK and total PER looks smooth, the peaks of free dCLOCK and total PER vary significantly, due to the transcription and translation noise. In Figure 2(c), dCLOCK.PER complex shows two peaks in one circadian cycle, because peaks of dCLOCK.PER are determined by peaks of both free dCLOCK and PER. Whether such dynamics reflect the level of dCLOCK.PER in real systems is still unknown experimentally [12].

**Figure 3:**
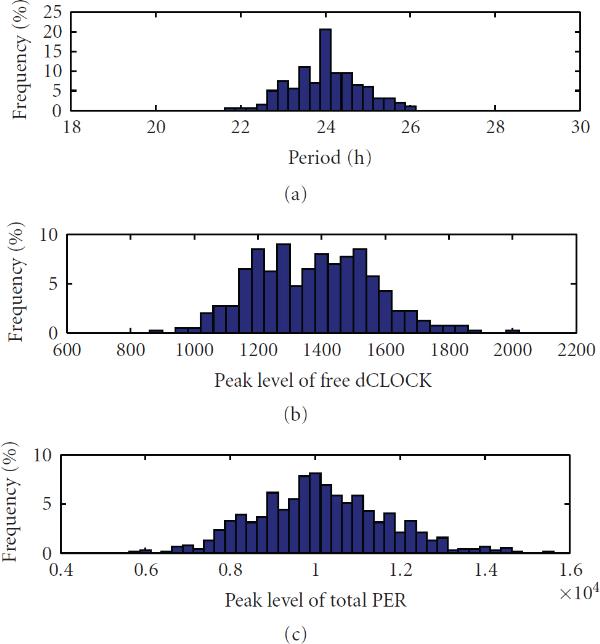
The histogram of periods and peaks of free dCLOCK and total PER for the detailed stochastic model in constant darkness.

We also simulated 100 runs to get the statistics of oscillation. Figure 3(a) depicts the histogram of oscillation periods. It is seen that most periods are within the range between 23 and 25 hours. Figures 3(b) and 3(c) show the histogram of the number of molecules of free dCLOCK and total PER at oscillation peaks, respectively. As listed in Table 4, the mean of the period is 23.93 hours, which is very close to 24 hours, and the SE of the period is 0.78 hours. The coefficient of variation (CV, SE divided by mean) is therefore , which is very low. Since CV is a normalized measure of dispersion of a probability distribution, a small CV for period implies that the periods lie in a small interval around its mean value with a large probability. Table 4 also contains the mean, SE and CV of the peak levels of free dCLOCK, total PER, and total dCLOCK, as well as the peak-to-through amplitude of total dCLOCK. Since the through amplitude of free dCLOCK and PER is zero, their peak-to-through amplitude is equal to their peak levels. It is seen that the CVs of the peak levels of free dCLOCK, total PER and total dCLOCK are , , , respectively, and that the CV of the peak-to-through amplitude of total dCLOCK is . Taken together, we see that the oscillation period is very robust in the presence of intrinsic noise, although there are significant fluctuations in oscillation peaks.

**Table 4:** Statistics of oscillations for the detailed stochastic model.

	Mean	SE	CV
Period (h)	23.93	0.78	3.26%
Peak value of total PER	10149	1530.1	15.08%
Peak value of free dCLOCK	1377.1	184.62	13.41%
Peak value of total dCLOCK	1437.1	156.24	10.87%
Peak-to-through amplitude of total dCLOCK	1016.5	191.51	18.84%

**Figure 4:**
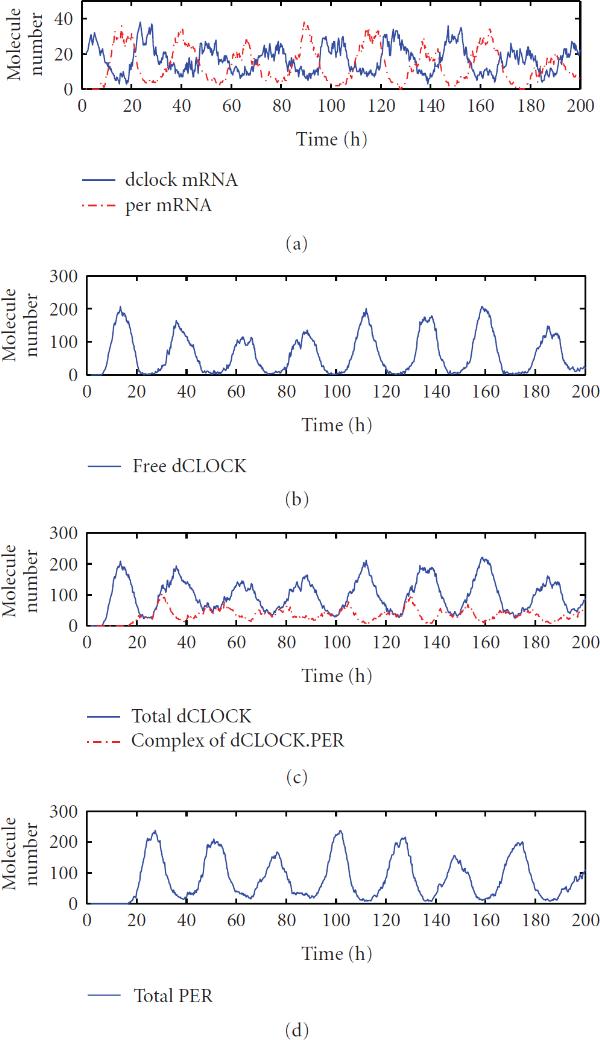
One trajectory of  and  mRNA, free dCLOCK, total dCLOCK, dCLOCK.PER complex, and total PER for the reduced stochastic model in constant darkness.

**Figure 5:**
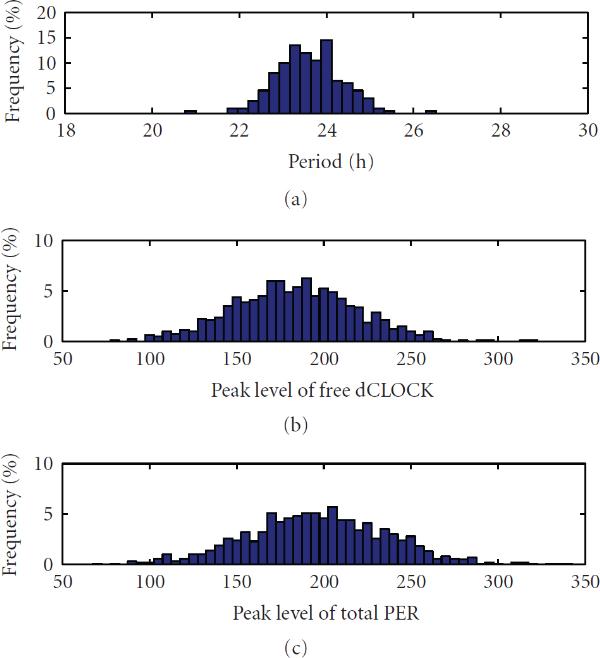
The histogram of periods and peaks of free dCLOCK and total PER for the reduced stochastic model under constant darkness.

We now discuss simulation results from the reduced model. Figure 4 shows one trajectory of  and  mRNA, free dCLOCK, total dCLOCK, dCLOCK.PER, and total PER. Again, consistent with the experiment observations,  and  oscillate in antiphase. Compared with the trajectories produced by the detailed model, the trajectories here appear to have more random fluctuations, which is due to the fact that the number of molecules of each species in the reduced model is much smaller than those of the detailed model. The histograms of periods and peaks of free dCLOCK and total PER peak obtained from 100 simulation runs are depicted in Figure 5. Table 5 lists the mean, SE and CV of the period, peaks of total PER, free dCLOCK, and total dCLOCK, as well as the peak-to-through amplitude of total dCLOCK. It is seen that the CV of period is almost the same as that of the detailed model, but the CVs of peaks and peak-to-through amplitude are slightly larger than those of the detailed model. Therefore, both the detailed and reduced models can produce robust oscillation period in the presence of intrinsic noise despite significant fluctuations in oscillation peaks. Note that levels of dCLOCK and PER are very different in two models. Therefore, our simulation results for two models demonstrate that oscillation is robust across a wide range of molecular levels or under quite different levels of intrinsic noise.

**Table 5:** Statistics of oscillations for the reduced stochastic model.

	Mean	SE	CV
Period (h)	23.60	0.80	3.39%
Peak value of Total PER	196.47	40.49	20.61%
Peak value of free dCLOCK	183.09	35.49	19.38%
Peak value of total dCLOCK	201.51	31.05	15.41%
Peak-to-through amplitude of total dCLOCK	172.47	36.10	20.93%

We investigate the effect of the random time delays with fixed average time delays. Since both detailed and reduced models produced similar results, we here only present results for detailed model. Note that time delays  and  in our simulations are random variables uniformly distributed in , where the standard value of  and standard value of , with  denoting the average time delay. To test the sensitivity of the range of random delays, we run more simulations using different  and  but with a fixed . Specifically, when we fix  to be 5 hours and 6 hours for  and , respectively, if  and  are uniformly distributed in , the mean period is 23.89 hours and the SE is 0.76 hour; if  and  are uniformly distributed in , the mean period is 23.81 hours and the SE is 0.79 hour. In both cases, the mean period and SE are very close to the results from standard value of  and . Therefore, our simulations show that the random changes in the delays do not cause significant variations in the oscillation period as long as the average delays are fixed.

Smolen et al. [12,14] also investigated the effects of noise using stochastic simulation. There are three major differences between our stochastic simulation and that of Smolen et al.: () we employed exact SSA, whereas they used approximate SSAs, () two delays critical to circadian oscillation are random in our simulation but deterministic in the simulation of Smolen et al., and () we explicitly simulated the transcription process and the binding/unbinding events between dCOLCK and per and dclock promoters, whereas Smolen et al. lumped transcription and translation of dclock and per into a one-step process.

To convert concentration into number of molecules, we used the volume of typical lateral neuron cells, whereas Smolen et al. determined a scale factor by trial. For the detailed model, this resulted in different scale factors and protein levels in our simulation as shown in Figure 2 are approximate 10 times of those in the simulation of Smolen et al. as depicted in Figure 3 of [12]. To make fair comparison, we ran simulations using the same scale factor as Smolen et al. [12]. Our simulation results showed that the mean peak values of PER, free dCLOCK and total dCLOCK are 1205, 176, and 183, respectively, which are comparable to the results of Smolen et al. [12]. The mean period in our simulation is 24 hours and the CV of periods is 3.33%. These results are also comparable to the results of Smolen et al.: a mean period of 23.5 hours and a CV of 5%. The CVs of the peaks of PER, free dCLOCK, and dCLOCK in our simulation are 15.47%, 15.20%, and 12.26%, respectively, which are greater than the CV of PER (9%) in the simulation of Smolen et al. [12]. For the reduced model, it turns out that protein levels in our simulation are similar to those in the simulation of Smolen et al. [14]. The CV of periods in our simulation (3.39%) is slightly smaller than that obtained in simulation of Smolen et al. (4.78%). Since no result about the CV of peak protein levels was reported by Smolen et al., we cannot compare the CV of peak protein levels.

In summary, although the noise in our models may be stronger than that in the models of Smolen et al. due to the random delays, transcription process, and random activation and repression of the promoters of per and dclock, the CV of periods in our simulation is slightly smaller than that in the simulation of Smolen et al. [14]. This result indicates that approximate simulation may have yielded nonnegligible errors. It is difficult to evaluate the effect of such possible errors in the approximate method of Smolen et al. [14], but our simulation method is exact and can correctly capture the stochastic dynamics of the circadian rhythm. It seems that strong noise in our model is reflected in the peak protein levels because the CVs of peak protein levels in our detailed model are larger than those in the detailed model of Smolen et al. [12].

### 3.2. Robustness Test in the Presence of Noise

In living cells, biochemical parameters often vary significantly from cell to cell due to stochastic effects, even if the cells are genetically identical [50]. But circadian oscillations with close period are still withstood in  or mammals. Therefore, a model of circadian rhythm should be robust in the sense that small parameter variations should not lead to large period variations. For the deterministic models, Smolen et al. [12,14] have shown that circadian rhythm is robust when a parameter changes its value by . Here, we test if circadian rhythm is robust with respect to parameter changes in the presence of intrinsic noise. To test robustness, each parameter is decreased or increased by  from the standard value, with all other parameters fixed at the standard values, and then the mean and SE of oscillation periods and peaks are determined from simulation results. Since  and  are random variables, we decrease or increase their mean values by .

**Figure 6:**
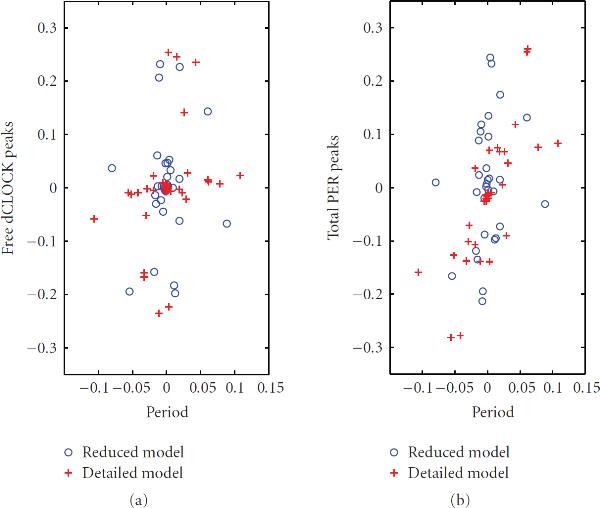
**Relative change of the mean values of periods and peaks of free dCLOCK (a) and total PER (b) after the value of one parameter increases or decreases by 20% of the standard value while other parameters are fixed**. The relative change of the period is defined as , where  is the mean of the period for the standard value of the parameter and  is for the new value of the parameter. The relative change of the peaks is defined similarly.

**Figure 7:**
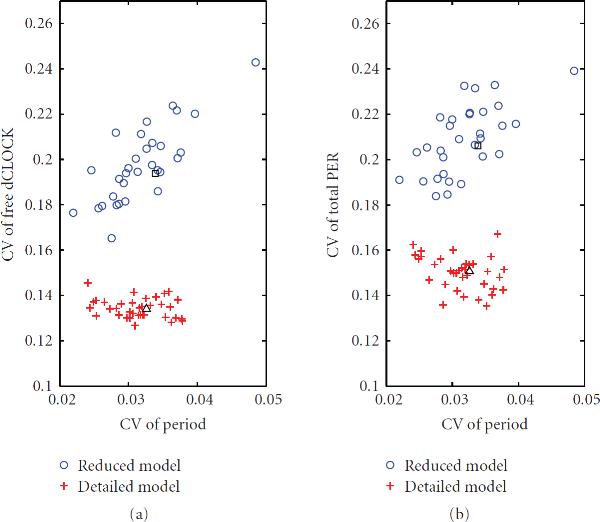
**CVs of periods and peaks of free dCLOCK (a) and total PER (b) after the value of one parameter increases or decreases by  of the standard value while other parameters are fixed.** CVs of periods and peaks of free dCLOCK and total PER for the standard parameter set are also shown as □ for reduced model and △ for detailed model.

We first tested the robustness of oscillations for the detailed model. There are 17 different probability rate constants and 2 time delays. Therefore, 39 set of simulations including the set with standard parameter values were run. Figure 6 plots the relative change of the mean values of periods and peaks between the results obtained using standard parameters and those obtained using a changed parameter. It is seen that most changes in the period are in the interval ( and that the changes in peaks are relatively large. Figure 7 plots the CV of the period and peaks for all parameter sets. It is seen that CV of the periods are very small, in the interval . When we changed each individual parameter by  of its standard value, the mean of the period was never changed more than . The period is most sensitive to , the time delay needed for  translation. When the mean value of  was decreased (increased) by  of its standard value, the mean period was decreased (increased) by  () and the CV of the period was  (), which is almost the same as the CV for the standard parameters. The peak of the free dCLOCK is most sensitive to , the probability rate constant of translation of  mRNA to dCLOCK protein. Decreasing (increasing)  by  decreased (increased) the mean peak of free dCLOCK by  (), and the corresponding CV was  (). The peak of the total PER is most sensitive to , the probability constant rate of translation of  mRNA to PER protein. Decreasing (increasing)  by  decreased (increased) the mean peak of total PER by  (), and the corresponding CV was  (). Therefore, the system appears to have small variation in the period but relatively large variation in the peaks when a parameter changes. This is very reasonable from the biological point of view since circadian rhythm is endogenous, which requires very small variation in the period even when some parameters are changed due to the change of external cues. The relatively large variation in the peaks is due to the stochastic fluctuation of gene transcription and translation.

We also tested the robustness of oscillation for the reduced model. The reduced model has 14 probability rate constants and 2 time delays. Therefore, 33 sets of simulations were run including the set with standard parameter values. Figure 6 plots relative change of the mean value of the period and peaks for the parameter sets with one changed parameter comparing with the standard parameter set and Figure 7 plots the CV of the period and peaks for all parameter sets. It is seen that the change is small in period but relatively large in peaks when a parameter changes. It is also seen that CV of the periods is very small for both models, in the interval . Therefore, the system is very robust to the parameter variation in oscillation period. Note that the CV of the peaks of the reduced model is larger than that of the detailed model. This is due to the fact that the reduced model has lower number of molecules in the system so that there is larger internal noise.

As in the detailed model, the period, the peak of the free dCLOCK and the peak of the total PER in the reduced model are most sensitive to , , and , respectively. Specifically, decreasing (increasing) the mean value of  by 20% decreased (increased) the mean period by  () and the corresponding CV was  (). Decreasing (increasing)  by  decreased (increased) the mean peak of free dCLOCK by  () and the corresponding CV was  (). Decreasing (increasing)  by  decreased (increased) the mean peak of total PER by  () and the corresponding CV was  (). Comparing the results of two models, it appears that both models have small changes in the mean period and relatively large changes in the mean peaks and that the reduced model has slightly larger CVs.

### 3.3. Light Entrainment of Oscillation in the Presence of Noise

Models of circadian rhythms must be able to maintain synchrony with environmental cycles to drive behavioral, physiological and metabolic outputs at appropriate time of day [7]. Circadian rhythms can be entrained by external cues, such as daily environmental cycles of light and temperature, but light is generally considered as the strongest and most pervasive factor. Therefore, the responses of the rhythm are often simulated by light pulses or light/dark (L/D) cycles [12,18,51–53]. We first consider the detailed model. In , light induces to enhance the degradation of phosphorylated TIM [12,54,55]. Since there is no separate variable for TIM in our model, the degradation of phosphorylated PER was induced to simulate the effect of light, as done by Smolen et al. [12]. Here the phosphorylated PER includes all unbounded and bounded PERs ( and dCLOCK. dCLOCK.). The dCLOCK is released after the PER complex with dCLOCK is degraded by light and the degradation rate of all phosphorylated PER is 0.9  [12]. In addition, to keep the oscillation period, the maximum degradation rate of dCLOCK, , was reduced to 1.5 nM  [12] and the probability rate constants  and ,  in our stochastic model were reduced correspondingly.

**Figure 8:**
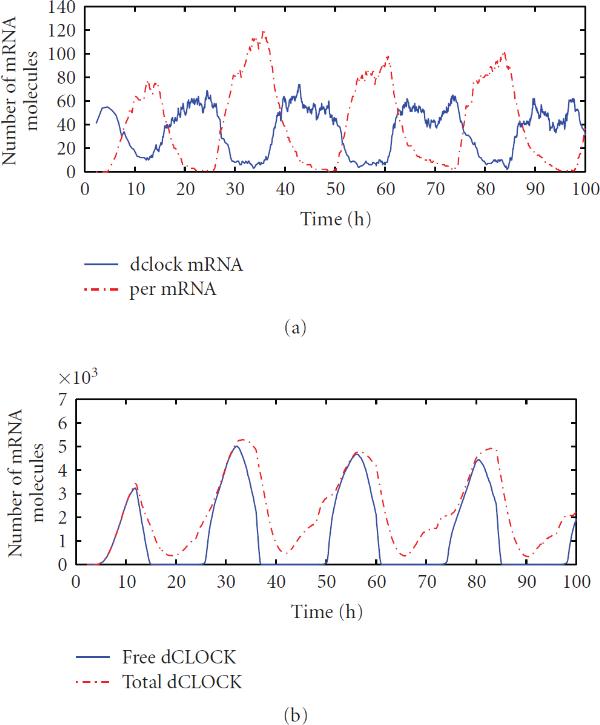
One trajectory of  and  mRNA, free dCLOCK and total dCLOCK protein for the detailed stochastic model with light response under L/D cycle.

**Figure 9:**
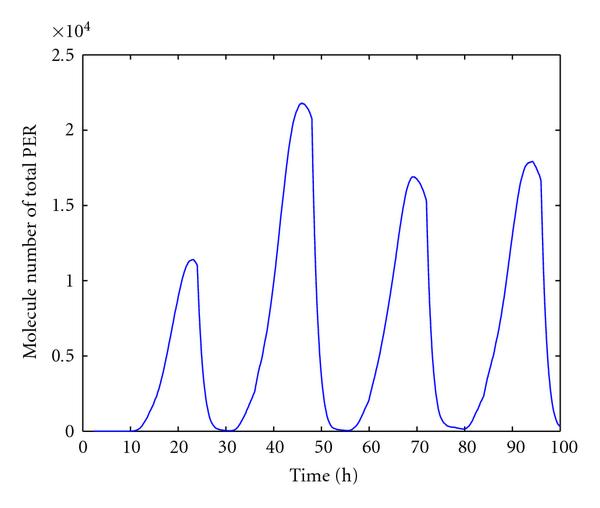
One trajectory of total PER protein for the detailed stochastic model with light response under L/D cycle.

Figures 8 and 9 plot one trajectory of  mRNA,  mRNA, free dCLOCK, total dCLOCK, and total PER, which demonstrates the entrainment of simulated circadian oscillations under the L/D cycle. The L/D cycle uses 12 hours light first and then 12 hours dark every 24 hours. It is seen that the peak of free dCLOCK is enhanced under new condition. Figure 9 also shows that the shape of the time trajectory of total PER under L/D circle differs significantly from that of the constant darkness as shown in Figure 2. The number of molecules of total PER drops off much more quickly when switching from dark to light than that in Figure 2, which is consistent with the experimental results [13,15] and Smolen's simulation results [12]. Moreover, the mean and CV of oscillation period obtained from 100 runs of simulation under the L/D cycle are  hours and , respectively. The mean and CV of peak values of free dCLOCK are  and , respectively. The mean and CV of peak value of total PER are  and , respectively. Therefore, the model not only runs well under the L/D cycle but also shows stable period but with considerable fluctuations in oscillation peaks.

**Figure 10:**
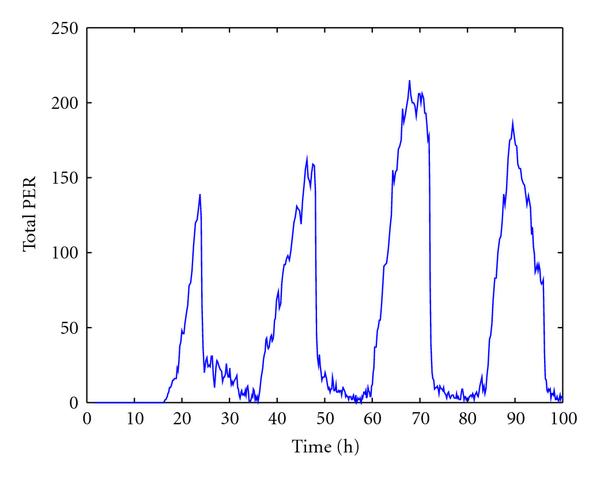
One trajectory of total PER protein for the reduced stochastic model with light response under L/D cycle.

L/D cycle was also applied to the reduced model to test the light entrainment. Since the light exposure was simulated by enhancing PER degradation [14], the probability rate constants for the degradation of unbounded PER and bounded PER,  and , both are increased by 4.5  [14]. Figure 10 shows one trajectory of total PER under L/D circle. Observations similar to those for the detailed model were seen: the number of molecules of total PER falls more quickly between dark-to-light switch than that under constant darkness; the oscillation appears to have a stable period but significant fluctuations in peak values of total PER as well as the peaks of free and total dCLOCK proteins (data not shown).

### 3.4. Impact of Transcription Activation Rate

As we mentioned earlier, the rate that dCLOCK binds to  and  genes is unknown but was estimated in our simulation. The rate that dCLOCK dissociates with  and  genes is chosen to be equal to the experimentally reported dissociation rate of myogenin protein with the E-box of E12 gene. In a deterministic model, these rates generally do not affect oscillation as long as their ratio is fixed. However, these rates may have significant effects on transcription noise even when their ratio is fixed [23,24,56]. In the following, we change the value of  while keep the ratio  fixed to see whether the oscillation period changes. Since both detailed model and reduced models yield similar results, we only give results for the detailed model.

**Figure 11:**
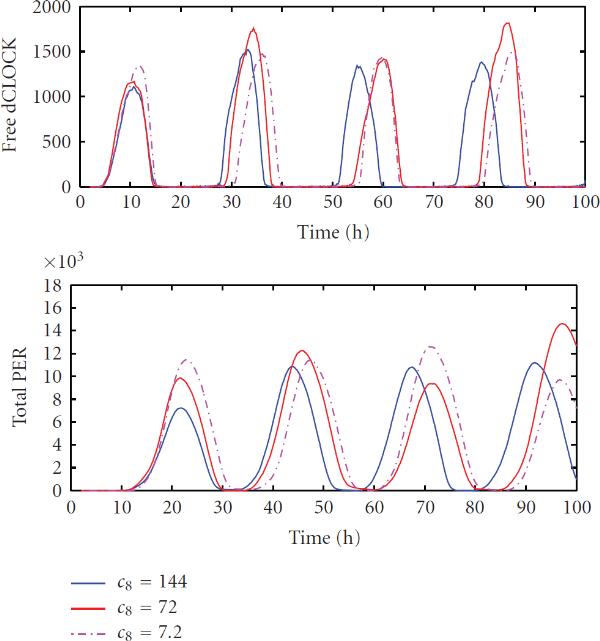
One trajectory of free dCLOCK and total PER protein for the detailed stochastic model with  , 72  and 7.2 .

The standard value of , chosen from experimental result, was 72  as described earlier and we ran simulations using two other values for : 144  and 7.2 . We found that if we further increase  beyond 144 , it would not affect simulation results. Therefore, we only compare the simulation results under these three values. Figure 11 shows one trajectory of free dCLOCK and total PER under three different values of . It is seen that the period of oscillations for the higher unbinding rate is slightly smaller than that for the lower unbinding rate. The mean values of the period obtained from 100 runs of simulation for , ,   were , , and  hours, respectively, and the correspondingly CVs are , , . The mean peaks of free dCLOCK, total dCLOCK, and total PER for three values of , as well as the corresponding CVs, are almost the same since the ratio of  is fixed. Therefore, under the three values tested, the rates that dCLOCK binds/unbinds to  and  genes do not have significant effect on oscillations as long as their ratio is fixed. However, if we further decrease the binding/unbinding rates by a factor of 100 and 1000, the mean of oscillation period changes to 26.70 hours and 37.40 hours, respectively. Note that this is consistent with the results of Forger and Peskin [56], as well as Gonze et al. [26], where oscillation period is changed significantly [56] or oscillations become irregular [26], when the binding and unbinding rates are decreased by at least two orders of magnitude. Since the rate change by a factor of 10 is significant, the oscillation period is relatively robust to the binding/unbinding rate within a reasonable range around the experimental reported rate.

## 4. Discussion

We have presented a detailed and a reduced stochastic model for delay-induced circadian rhythm in  based on the deterministic models of Smolen et al. [12,14], and employed our recently developed exact stochastic simulation algorithm [33] to simulate the circadian rhythm. This work is unique since no exact stochastic simulation has been carried out for circadian rhythms based on a model with random time delays. As discussed in [33], several SSAs have been developed for reaction systems with delays [57,58]. However, the algorithm in [57] and two algorithms in [58] are not exact. The work in [33] also proved that another heuristic algorithm in [58] is exact but requires more computation than the exact SSA in [33]. Since both algorithms are exact, they should produce the same statistical results. Another SSA for systems with delays was proposed in [59], but an approach similar to that in [57] was used, and thus, it is not exact either. Smolen et al. [12,14], as well as Li and Lang [32], also simulated delay-induced circadian oscillation, but they used approximate stochastic simulation methods.

Our simulation results demonstrated that the intrinsic noise causes large fluctuations in oscillation peaks but very small fluctuations in oscillation period. This observation is seen in all simulations under different conditions, such as constant darkness and L/D cycles. Deterministic simulation cannot reveal this phenomena, since both period and peaks are constant. Our stochastic simulations also showed that circadian oscillation is robust in the presence of noise in the sense that noise has little effect on oscillation period although it can change oscillation peaks significantly. We also showed that random delays within certain range do not cause significant variations in the oscillation period as long as the average delays are fixed. To the best of our knowledge, these two results have not been observed in previous stochastic simulation of circadian rhythms. These two observations imply that circadian oscillation is robust in the presence of noise and random delays and that the randomness inherent to the oscillation circuit may not have much biological impact on the organism. As discussed in [50], when a protein regulates its targets, it often operates on a Hill curve. Once the level of the regulating protein is higher or lower than certain value, the protein operates at the top or bottom of the curve and the fluctuation of its level to certain extend does not affect much the regulating effect on its targets. Therefore, the relatively large variations in the peak values of PER and dCLOCK proteins observed in our simulation may not have a strong biological impact.

Similar to previous deterministic simulations and approximate stochastic simulations [12,14], our stochastic simulation shows that both detailed and reduced stochastic models can provide sustained oscillations under darkness and L/D cycles. Our results also show right phase of all the components in the system, correct phase and anti-phase relationship of mRNAs and proteins, and also the appropriate lags between mRNAs and proteins. Our stochastic simulation further demonstrated that circadian rhythm is robust to parameter variations in the presence of noise. Increasing or decreasing each parameter by  of its standard value changes the mean period by less than  and causes negligible changes in the CV of oscillation periods. The model is not sensitive to the time delay during the  mRNA translation, but it is most sensitive to the average time delay during  mRNA translation, which shows that time delay is essential to circadian oscillation in the two models. However, random fluctuations in these two time delays have little effect on the oscillation period as long as the average delays are fixed. We also found that the binding and unbinding rates of dCLOCK to  and  genes within a reasonable range have little impact on the circadian oscillation. Increasing or decreasing the binding and unbinding rates by 10 times relative to an experimentally reported rate while keeping their ratio fixed does not cause significant changes in the period and peaks of oscillation.

We have compared our exact simulations with approximate simulations of Smolen et al. [12,14] in Section 3.1. Another work by Li and Lang [32] also employed approximate SSAs to simulate the reduced model of Smolen et al. [14]. Like Smolen et al. [12,14], Li and Lang [32] used deterministic delays, whereas we employed random delays which are more appropriate to reflect the delays in transcription, translation, and other chemical process. Li and Lang emphasized on the noise induced oscillation and showed that noise can sustain oscillation in the parameter region where no oscillation is predicted by the deterministic model, whereas we here focused on the robustness of oscillation in the presence of intrinsic noise and the effect of random delays. We showed that the oscillation is robust in the presence of noise since there is very little variability in oscillation period in spite of large random variability in peaks, and that random changes in delays within a large interval around the fixed average delay cause little variability in the oscillation period.
